# AI in Radiology Has Come to Stay

**DOI:** 10.1007/s00062-026-01638-4

**Published:** 2026-03-02

**Authors:** Martin Bendszus

**Affiliations:** Abteilung für Neuroradiologie, Universitätsklinikum Heidelberg, Heidelberg, Germany

The 2025 annual meeting of the Radiological Society of North America (RSNA) left little doubt: artificial intelligence (AI) has moved from innovation showcase to clinical infrastructure. Nowhere was this more evident than in neuroradiology. AI is no longer a peripheral research topic—it is becoming embedded in acquisition, reconstruction, triage, quantification, and reporting.

Many presentations at the RSNA showed a table shift compared with previous years with a transition from retrospective proof-of-concept studies to prospective, multicenter validation and workflow-integrated solutions. Deep learning-based MRI reconstruction techniques presented at RSNA demonstrated accelerated acquisition times—often reduced by up to 50%—while maintaining or improving image quality and lesion conspicuity, consistent with earlier multicenter validation data (e.g., [1]). In acute stroke imaging, AI-assisted detection of large vessel occlusion and automated perfusion analysis were associated with improved door-to-treatment metrics in real-world settings, echoing findings reported by Amukotuwa et al. [2] and subsequent single center implementation studies.

In neuro-oncology, automated longitudinal tumor segmentation and volumetric analytics were validated across institutions, including federated learning strategies designed to enhance generalizability while preserving data privacy. The maturation of these approaches aligns with broader trends in additional quantitative imaging biomarkers complementing recent consensus statements by the Quantitative Imaging Biomarkers Alliance (QIBA, [3, 4]).

Another key theme was the emergence of multimodal foundation models integrating imaging, clinical variables, and molecular information. Early prospective data suggest that such systems may support non-invasive glioma characterization and structured reporting augmentation. Importantly, the dominant paradigm is not automation but augmentation: AI as a cognitive partner enhancing reproducibility, efficiency, and quantitative rigor and not replacing radiologists assessment.

Equally prominent were discussions on bias, regulatory oversight, interoperability, and medico-legal implications—underscoring that technical performance alone is insufficient without robust validation, transparent governance and legal compliance.

For neuroradiology, the implications are clear. Quantitative imaging biomarkers are transitioning from research tools to clinical standards. Prospective outcome studies must accompany technological advances to prove patient benefit by these new techniques. And AI literacy must become integral to training and continuing education.

RSNA 2025 did not merely showcase better algorithms. It marked the consolidation of AI as an integral component of neuroradiological practice. AI has entered our clinical practice and it has come to stay.
